# Profile formation of academic self-concept in elementary school students in grades 1 to 4

**DOI:** 10.1371/journal.pone.0177854

**Published:** 2017-05-18

**Authors:** Isabelle Schmidt, Martin Brunner, Lena Keller, Vsevolod Scherrer, Rachel Wollschläger, Tanja Gabriele Baudson, Franzis Preckel

**Affiliations:** 1 Department of Psychology, University of Trier, Trier, Germany; 2 Department of Educational Sciences, University of Potsdam, Potsdam, Germany; 3 Department of Education and Psychology, Free University of Berlin, Berlin, Germany; 4 Faculty of Language and Literature, Humanities, Arts and Education /Luxembourg Centre for Educational Testing (LUCET), University of Luxembourg, Esch-sur-Alzette, Luxembourg; 5 Faculty of Rehabilitation Sciences, TU Dortmund University, Dortmund, Germany; IUMPA - Universitat Politecnica de Valencia, SPAIN

## Abstract

Academic self-concept (ASC) is comprised of individual perceptions of one’s own academic ability. In a cross-sectional quasi-representative sample of 3,779 German elementary school children in grades 1 to 4, we investigated (a) the structure of ASC, (b) ASC profile formation, an aspect of differentiation that is reflected in lower correlations between domain-specific ASCs with increasing grade level, (c) the impact of (internal) dimensional comparisons of one’s own ability in different school subjects for profile formation of ASC, and (d) the role played by differences in school grades between subjects for these dimensional comparisons. The nested Marsh/Shavelson model, with general ASC at the apex and math, writing, and reading ASC as specific factors nested under general ASC fitted the data at all grade levels. A first-order factor model with math, writing, reading, and general ASCs as correlated factors provided a good fit, too. ASC profile formation became apparent during the first two to three years of school. Dimensional comparisons across subjects contributed to ASC profile formation. School grades enhanced these comparisons, especially when achievement profiles were uneven. In part, findings depended on the assumed structural model of ASCs. Implications for further research are discussed with special regard to factors influencing and moderating dimensional comparisons.

## Introduction

Academic self-concept (ASC) is comprised of mental representations of one’s abilities in academic subjects. ASC plays an important role in educational psychology because it influences scholastic achievement (e.g., [1]), academic motivation and affect [2], and educational choices [3]. Understanding the development of ASC across the school career and its determinants is therefore an important issue.

The investigation of ASC is intrinsically tied to theories about its structure. Research on the structure of ASC demonstrated that, from age 4 onward, ASC is a multidimensional construct with separate, domain-specific mental representations of ASCs for various achievement domains (such as specific school subjects) (e.g., [4–6]), a finding that generalizes across countries (e.g., [7–8]). Further, subject-specific ASC is comprised of a cognitive and an affective component (e.g., cognitive: “Math is one of my best subjects”; affective: “I like math”). However, in line with most research on ASC (e.g., [9]), the present study focuses on the cognitive component. Besides the multidimensionality of ASC, there is evidence for a general ASC, which can be assessed by items such as “I am a good student.” Nonetheless, exactly how general ASC should be incorporated into the structural model of ASC and at which hierarchical level remains a matter of debate. This is crucial for research questions focusing on a key aspect of the differentiation of ASC, that is, the formation of the profile of one’s own strengths and weaknesses. For example, one student may have a higher general ASC than another student. Still, both students may think they are better in math than in their native language. That is, despite mean level differences, both students can share a similar ASC profile.

The most recent structural model of ASC, the nested Marsh/Shavelson model (NMS model) ([9]; see also [10–11]), seems especially appropriate for tackling questions on the profile formation of ASC because it allows to control for the general ASC level in students’ profiles, thus directly depicting students’ ASC profiles of subjective strengths and weaknesses in particular subjects. Further, the NMS model accounts for the shape of students’ profiles. There is broad empirical support for the NMS model of ASC in secondary school students [9–11]. However, to our knowledge, no studies have yet examined how well the NMS model of ASC captures the different facets of ASC in elementary school students.

To understand ASC profile formation, it is important to take different sources of information into account. It is well documented that ASC are formed on the basis of multiple sources, such as feedback from significant others, and against multiple frame of references [12]. The internal/external frame of reference model (I/E model; [13]) posits that students mainly use an external (social) and an internal (dimensional) frame of reference to evaluate their subject-specific ASC. Within a student’s dimensional frame of reference, the student compares his or her achievements across subjects, and this is assumed to be a central source for ASC profile formation [14]. Most support for the I/E model stems from secondary school students [15]; a few studies also found support for it in samples of elementary school students from grade 3 onwards (e.g., [15–17]). However, none of these studies used the NMS model of ASC although, as outlined above, it is particularly well suited for investigating the impact of dimensional comparisons on profile formation.

Moreover, research on variables that moderate the effect of dimensional comparisons is still rare [14]. Rost et al. [18–19] identified the difference between math and native language grades as a moderator. However, Rost et al. drew on data from secondary school students only; it therefore remains unclear to what extent these findings also apply to elementary school students.

In sum, whereas the structural models of ASC and the factors influencing ASC profile formation are well studied in adolescents, research on elementary school students is comparably scarce. Expanding our knowledge to younger students is crucial, for example, to understand the development of ASC in this age group and to implement early interventions to support the positive development of ASC.

The purpose of this article is to fill this gap. In a large quasi-representative cross-sectional sample of German elementary school students in grades 1 to 4, we investigated (a) the profile formation of ASC using alternative structural models of ASC; (b) the impact of external and dimensional comparisons within the I/E model; and (c) the moderating effect of differences in teacher-assigned grades in the subjects math and German on the strength of dimensional comparisons. To this end, we applied the NMS model and alternative structural models of ASC and systematically compared the results for these models.

### Different approaches to model the structure of academic self-concept

**Fig 1:**
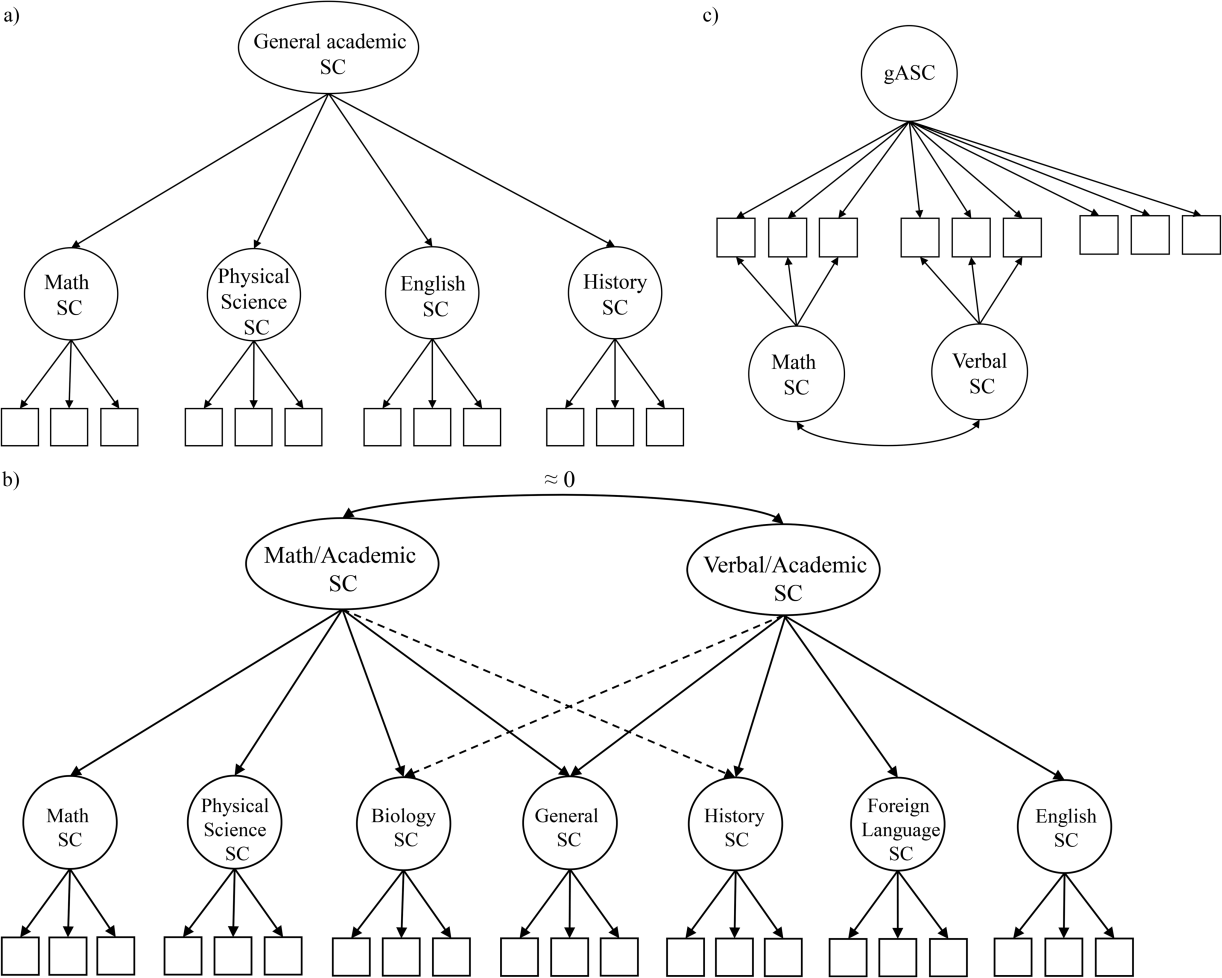
Structural conceptions of academic self-concept (ASC). **(a) The academic section of the original model by Shavelson et al. [20], (b) an elaboration of the Marsh/Shavelson model, (c) an elaboration of the nested Marsh/Shavelson model [11]. (a) and (b) based on Marsh et al. [22].** gASC = general ASC; SC = self-concept. To ensure clarity of presentation, residual terms of the manifest variables were omitted.

Shavelson, Hubner, and Stanton [20] posited self-concept as a multidimensional (multifaceted) and hierarchical construct, with substantially correlated different facets of ASC forming a single higher-order factor representing general ASC (Shavelson model; see Fig 1(A)). The multidimensionality of ASC can be depicted within a first-order correlated factor model in which the different subject-specific ASC are distinct but correlated factors. However, subsequent research showed that within the first-order correlated factor model of ASC, math ASC and native language (verbal) ASC were nearly uncorrelated. This finding was incompatible with the assumption that a single higher-order factor accounts for the correlation between the first-order factors. Thus, the Shavelson model was revised, leading to the Marsh/Shavelson model (see Fig 1(B); [21]; see also [22]). This model comprises two higher-order ASC factors (math/academic and verbal/academic) instead of one general higher-order factor. Moreover, general ASC is subordinate and influenced simultaneously by the two higher-order ASC factors. Several studies identifying positive correlations of general ASC with both native language and math ASC (e.g., [13, 23]) led Brunner et al. [9–11] to propose a new structural model of ASC, which they called the nested Marsh/Shavelson model (NMS model; Fig 1(C)). The NMS model combined the assumption of a general ASC at the apex and the multidimensional nature of ASC. In this model, general ASC (gASC) exerts a direct influence on all indicators of ASC and its facets—that is, general as well as all subject-specific measures. Hence, gASC is the most general factor in the NMS model, which is in line with its original conception in the Shavelson model [20]. The subject-specific ASC factors (e.g., subject-specific math ASC or subject-specific verbal ASC) are nested under gASC and represent the multidimensional nature of ASC. In the NMS model, gASC is uncorrelated with the subject-specific ASCs, whereas the subject-specific ASCs are allowed to correlate. Thus, both gASC and subject-specific ASC explain incremental variance in subject-specific ASC measures such that their variability is decomposed into a part shared with gASC and a part unique to each subject-specific ASC. Thus, subject-specific ASC factors are residualized while the variance attributable to a gASC factor is controlled for. The correlation between the different subject-specific ASCs indicates how students contrast their strengths and weaknesses in various subjects against each other, thereby providing an insight into their profile formation. Given that gASC is controlled for in measures of subject-specific ASC, the NMS model allows researchers to investigate the profile formation of subject-specific ASC independent of the general ASC level. For example, previous research on the NMS model consistently found negative correlations between subject-specific ASC in math and verbal domains, which demonstrates that people think of themselves as either “math” or “verbal” persons (e.g., [9–11]; see also [7]). Notably, the NMS model usually comprises indicators for general ASC but does not include a specific (nested) factor for these indicators. Unlike the subject-specific indicators, the general indicators therefore load on one factor only (i.e., gASC). Hence, the model specification of the NMS model has the advantage that gASC, as the general factor, is psychometrically well defined by these indicators, which implies that the substantive interpretation of gASC as well as the correlational patterns between subject-specific ASC factors do not depend on the number of academic subjects investigated (see [24–25]).

Taken together, compared to a first-order correlated factor model depicting the multidimensional nature of ASCs only, the NMS model accounts for the multidimensional nature and the hierarchical structure of ASC. In a representative sample of Luxembourgish 8th graders, the NMS model described the structure of ASC well, and better than the hierarchical Shavelson model with one second-order factor of general ASC or the Marsh/Shavelson model [9]. In a cross-cultural sample of 15-year-old students in 26 countries, a restricted version of the NMS model using measures of general ASC, math ASC, and verbal ASC accounted well for the structure of ASCs [10]. Yet up to now, the NMS model has never been investigated in elementary school students.

### Profile formation of academic self-concept in elementary school students within the framework of different structural models

Young children usually report unrealistically high levels of ASC, which has been explained by their lower abstract thinking skills, compared to older children [26]. With cognitive development and environmental changes, reports of ASC become more realistic such that the mean ASC level decreases with age, and ASC correlates more strongly with external indicators of competence [6, 27–29]. In particular, this development relates to two abilities: the ability to understand, interpret, and integrate ability-related experiences (such as feedback from significant others, e.g., teachers, parents, peers), and the ability to integrate information from social comparisons into one’s ASC (as well as the increasing use of such information with age) ([30]; see [31] for a summary). Furthermore, environmental changes (e.g., increasing competition) and more salient feedback practices (e.g., grading) contribute to the development of a more realistic ASC during elementary school [31]. In addition, an individual’s grasp of self-concept changes from concrete descriptions of behavior in early childhood to trait-like psychological constructs in middle childhood, and to more abstract constructs during adolescence [26]. From middle to late childhood, children are able to integrate opposing but coexisting concepts into their self-concept (e.g., to be dumb and smart at the same time). During late childhood, comparisons shift from those between different areas (e.g., students, friend) to comparisons between domains within the same area (e.g., in school: comparison of one’s own ability in different school subjects), and to comparisons within a single domain (e.g., reading and writing in one’s native language). Therefore, with increasing age, dimensional comparison processes become more differentiated and more relevant for children’s views of themselves [26]. Studies investigating the differentiation of self-concept showed that from age 4 to 5 onwards, children were able to discriminate between the different facets of ASC (e.g., reading ASC and math ASC)[4,32]). Summarizing the findings from several studies using the Self Description Questionnaire (SDQ-I; [33]), Marsh [12] concluded that correlations between math ASC and reading ASC were substantial in grades 2 and 3 (*r*s = .46/.47) but low from grade 4 onwards. Besides comparing the relationship between the different ASC, Marsh [27] found evidence for the differentiation of the different facets of ASC from grades 2 to 4, but not beyond. Furthermore, previous research indicates that whereas the correlation between math and reading ASCs decreased over time, the correlations between general ASC and math or reading ASC were moderate in size and remained relatively stable across different ages (e.g., [5, 32]).

One possible explanation for these complex age-related changes of correlational patterns among the different facets ASCs is Marsh and Ayotte’s differential distinctiveness hypothesis [34]. It predicts an increasing differentiation of disparate areas of ASC but also an increasing integration of closely linked areas of ASC with age and cognitive development ([34], p. 689). Marsh and Ayotte summarized that “self-concept factors that were more closely associated in the self-concept hierarchy (e.g., those associated with the same higher-order factor) were predicted to show the least (or no) decline in the sizes of the correlations as age increased” ([34], p. 691). The differential distinctiveness hypothesis is provided by cross-sectional data spanning grades 2 to 6 [34]. Specifically, the correlation between reading and math ASC, which pertains to different higher-order factors in the Marsh/Shavelson model of ASC, was lower in older than in younger children, whereas their correlations with general ASC remained relatively stable. However, Marsh and Ayotte [34] made no assumptions about changes in the correlation among the facets of skill-specific ASC of the verbal ASC, such as reading ASC and writing ASC.

A few studies investigated the hierarchy within and multidimensionality of verbal ASC in samples comprising students from secondary school and beyond (e.g., [35–38]). They found evidence for a hierarchical and multidimensional verbal ASC, with a general verbal ASC as a higher-order factor as well as skill-specific verbal ASCs (i.e., reading, writing). For eighth- and ninth-grade students as well as for university students, correlations between different skill-specific verbal ASC were high (*r*s ranging between .53 and .87). Following Marsh and Ayotte’s train of thought that the relationship between self-concepts belonging to the same higher-order factor would strengthen with age [34], the correlation of reading ASC and writing ASC, as skill-specific self-concepts of the verbal domain, should at least be stable or even increase with age. To date, this has not been explicitly tested.

In general, reading ASC has been examined most frequently as a single indicator of verbal ASC. In contrast, writing ASC has received comparably little attention in research on the structure of ASC, although research has indicated that reading ASC does not cover verbal ASC sufficiently [37]. In elementary school education, both reading and writing are core skills that students have to master [16]. Previous research has found reading ASC and writing ASC to be distinct but correlated factors within verbal ASC. For instance, Ehm et al. [16], examining third graders, showed that a first-order correlated factor model of ASC with writing ASC, reading ASC, and math ASC provided a better fit to the data than a model with one combined verbal ASC factor. In this study, the correlation between reading ASC and writing ASC was substantial (*r* = .66). Poloczeck, Karst, Praetorius, and Lipowsky [39] found a similar correlation between reading ASC and writing ASC (*r* = .58) for first grade elementary students. Taken together, these findings indicate positive correlations of similar size between reading ASC and writing ASC in samples of children, adolescents, and adults.

It should be noted that, for the most part, previous research investigating the differentiation of self-concept has compared the sizes of correlations between different self-concept facets using factor scores from first-order confirmatory factor analysis, factor scores from exploratory factor analysis, or manifest scale scores. However, in these studies, the overall level of subject-specific ASC confounds variability unique to the subject-specific ASC and variability due to general ASC. When investigating students’ ASC profile formation as one key aspect of ASC differentiation, it is advantageous to use factor scores derived from the NMS model, which do not suffer from this confound because the general level of ASC is controlled for in subject-specific measures (see Fig 1(C)). Thus, differentiation in students’ profiles of subject-specific ASC is represented more clearly in the NMS model than in the first-order correlated factor model, where the two sources of variance are not separated so that subject-specific ASC factors still contain both. Therefore, correlational patterns among the different subject-specific ASCs are more ambiguous to interpret because changes in these correlations may be attributable to (a) changes in students’ ASC profile or (b) changes in the relation of subject-specific measures to gASC, that is, the amount of variability in subject-specific ASCs explained by general ASC.

To conclude, no study has yet investigated profile formation of ASC within the NMS model of ASC based on the assumptions of the differential distinctiveness hypotheses considering the different subject-specific ASCs as well as skill-specific ASC in elementary school students. Available research findings suggest that with increasing age, disparate areas of ASC (e.g., reading ASC and math ASC) correlate less, whereas correlations between closely linked areas of ASC (e.g., reading ASC and writing ASC) remain stable or even increase.

### The internal/external frame of reference model in elementary school students and the role of achievement differences across domains in the I/E model

The internal/external frame of reference model (I/E model; [13]) states that external and internal (dimensional) comparison processes play an important role in the formation of ASC. First, students make use of external comparisons: They compare their performance in a particular subject with the performance of other students in the same subject and with further external standards of actual achievement level. Second, students make use of dimensional comparisons, which occur when students contrast their performance in one particular school subject with their performance in other subjects. External comparisons lead to positive correlations between achievement and the corresponding ASC within a domain, whereas dimensional comparisons can either reduce correlations between subject-specific ASCs through contrast effects or increase correlations through assimilation effects. Typically, the assumptions of the I/E model are tested via path models. In a path model, the effects of external comparisons are evidenced by positive paths from achievement to the student’s ASC in the corresponding subject. For dimensional comparisons, assimilation effects are evidenced by positive paths from achievement in one subject to the ASC in the noncorresponding subject. Contrast effects, on the other hand, are evidenced by negative paths between achievement and ASC of noncorresponding subjects. Summarizing previous research on dimensional comparison processes, Möller and Marsh’s [14] dimensional comparison theory posits that contrast effects are more likely when subjects are dissimilar. Based on the similarity/dissimilarity of subjects, Marsh et al. [40] arranged the subject-specific ASCs (e.g., math ASC, biology ASC, or foreign language ASC) along a continuum that places ASCs of similar subjects close to each other (see Fig 1(B)). Math and verbal (reading/writing) ASCs as the most dissimilar subjects are located at the opposite ends of this continuum. Contrast effects are more likely between “distant” subjects, whereas assimilation effects are more likely between “close” subjects or subjects pertaining to the same domain (e.g., reading/writing). Thus, comparisons of achievement in math and native language (e.g., reading/writing) should result in the largest contrast effects.

Most studies have investigated the I/E model with the subjects math and native language. For this case of subjects from dissimilar domains, cross paths leading from math achievement to verbal ASC and vice versa are predicted to be negative (i.e., contrast effects). A large number of studies on math and verbal subjects support the I/E model. However, most of those studies have focused on secondary school students. In their meta-analysis of I/E model findings, Möller et al. [15] noted that elementary school students were examined in only 3 out of 69 samples. Moreover, none of these 3 studies covered both reading and writing ASC, although previous research showed the two to be separate factors of verbal ASC (see above). Hence, in more recent studies, the I/E model was extended. For instance, Ehm et al. [16] investigated the I/E model with reading ASC, writing ASC, and math ASC in elementary school grades 1, 2, and 3. They found evidence for dimensional comparisons only in 3rd grade, and this for reading and math only. Poloczeck et al. [39] examined the I/E model with math and reading in first-graders and also found no effects of dimensional comparisons for this grade level. These findings indicate that effects of dimensional comparisons are likely to occur from 3rd grade onwards.

Most I/E studies have either neglected the general ASC factor in favor of domain-specific ASC factors or included a general ASC and a general achievement indicator as correlated factors to control for their influence while studying the I/E model (e.g., [13]). An exception is a study by Brunner and colleagues ([9]; see also [11]) which incorporated general ASC using the NMS model and contrasted the results with the common test of the I/E model. Across both ASC models, the effects of external and internal comparisons assumed by the I/E model could be supported. However, no study has yet modeled the assumptions of the I/E model by applying the NMS model to elementary school students.

Marsh ([41], p. 110) framed the predictions of the I/E model as general effects because lower self-perceived skills in one's native language will lead to higher levels of math ASC for students across the entire math skill spectrum and vice versa. However, these effects may be reinforced by an interaction of achievement in both subjects. That is, the effect of internal comparisons may increase when achievement differences in contrasting subjects become larger and thus more salient. For example, Rost et al. [18] (see also [19]), using a quasi-experimental approach, found that effects of internal comparisons were stronger when achievement levels diverged in the different subjects. In a sample of German students (7th/8th grade), Rost et al. categorized students into different groups: students with identical grades in all subjects (Group 1), students with a one-grade difference between subjects (Group 2), and students with a greater-than-one grade difference between subjects (Group 3). In Group 1, the correlation between the different subject-specific ASCs was substantial and positive, whereas it was lower in Group 2, and lowest in Group 3. Whether this moderating effect of achievement differences for dimensional comparisons generalizes to students in elementary school remains an open question.

### Research aims of the present study

**Fig 2:**
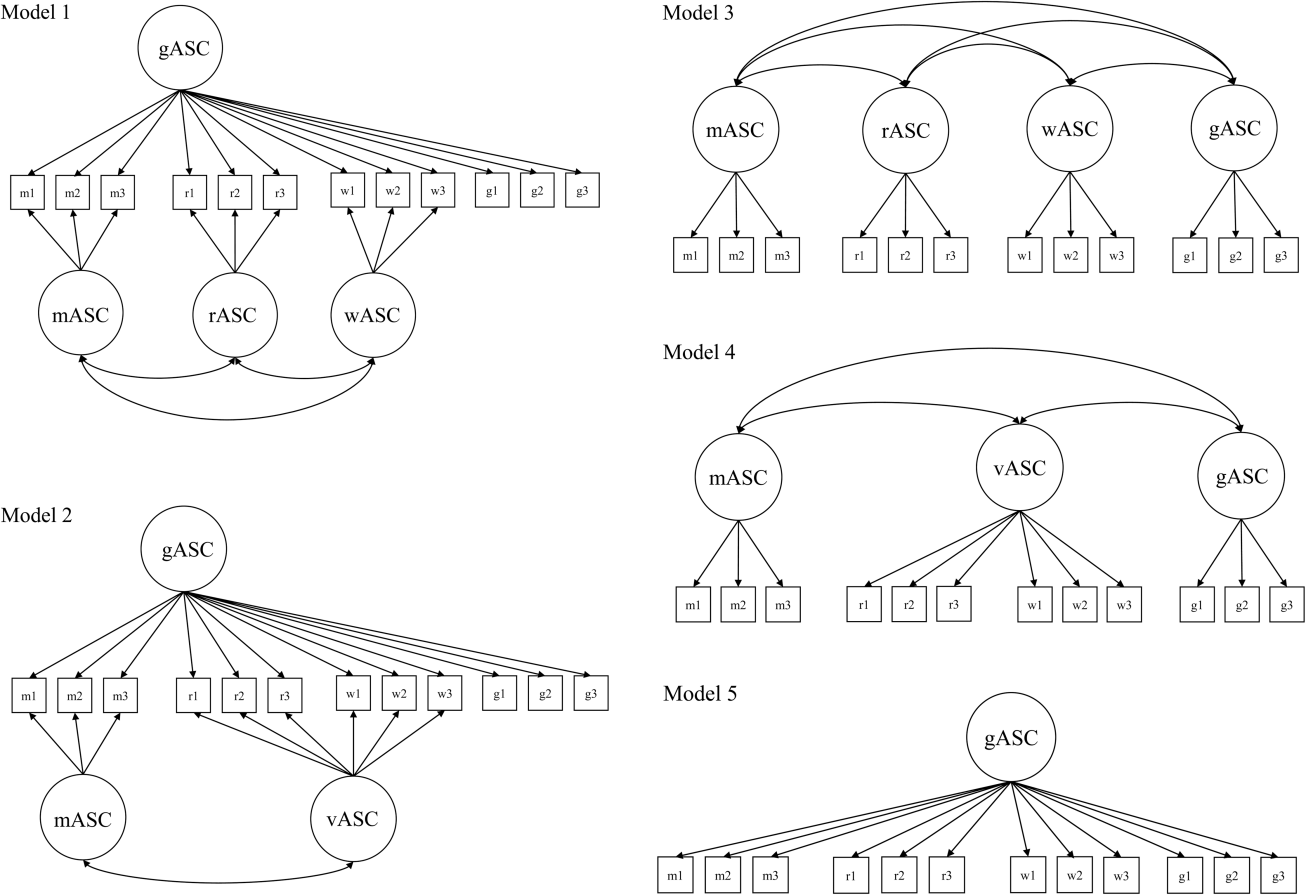
**Model 1: NMS_3sf = Nested Marsh/Shavelson model of academic self-concept (ASC) with math ASC, writing ASC, and reading ASC as correlated specific factors. Model 2: Nested Marsh/Shavelson model of ASC with math ASC and verbal ASC as correlated specific factors. Model 3: FOCF_4f = First-order correlated factor model with general ASC, math ASC, reading ASC, and writing ASC. Model 4: FOCF_3f = First-order correlated factor model with general ASC, math ASC, and verbal ASC. Model 5 = *g*-factor model in which all ASC items load on one factor.** gASC = general ASC, mASC = math ASC, wASC = writing ASC, rASC = reading ASC, vASC = verbal ASC. For the sake of clarity, residual terms of the manifest variables were omitted.

In the present study, we capitalized on data from a quasi-representative cross-sectional sample of German elementary school students in grades 1 to 4. We pursued three major research goals:

First, we tested the suitability of the NMS model of ASC in elementary school students in comparison to other structural models. For this age group, there is evidence for a general ASC, for different subject-specific ASCs [13], and for distinct writing and reading factors of verbal ASC [16, 36]. Therefore, an NMS model of ASC [10] with math ASC, writing ASC, and reading ASC as three correlated specific factors nested under a general ASC (gASC; Model 1) should provide a good fit to the data of elementary school students. Furthermore, a first-order correlated factor model with general ASC and math ASC, writing ASC, and reading ASC should fit the data well, too (Model 3). For the sake of completeness, we compared these two models to the following models: an NMS model with reading and writing collapsed into a single verbal ASC factor (Model 2); a first-order correlated factor model with general ASC, math ASC, and one verbal ASC factor (Model 4); a *g*-factor model in which all indicators (i.e., items) of general ASC, math ASC, reading ASC, and writing ASC were explained by one first-order factor (Model 5). All models tested in this study are shown in Fig 2.

Second, we investigated how ASC profiles form in elementary school students. We assumed profile formation to take place early in elementary school (from middle to late childhood; e.g., [26, 29]). In line with the differential distinctiveness hypothesis [34], we expected to find (a) an increasing integration of closely related areas of self-concept (e.g., writing/reading ASC), resulting in stable or increasing positive correlations with increasing grade level. Further, we expected to find (b) a differentiation of diverging areas of self-concept (e.g., math/writing ASC and math/reading ASC), resulting in decreasing positive correlations moving to zero or becoming negative and increasing in size with increasing grade level. Previous studies mainly used a first-order correlated factor model of ASC to investigate this research question. To align our results with those from previous research, we investigated profile formation of ASC both within the first-order correlated factor model of ASC and within the NMS model (see Fig 2: Model 1 and Model 3). In the NMS model, variability in subject-specific ASC measures is residualized by controlling for the variance attributable to gASC. Accordingly, correlations among subject-specific ASCs (“specific factors”) directly depict the profile formation of subject-specific ASCs independent of the general level of the ASC profile. Therefore, we expected the change in correlations between the domain-specific ASCs to become more apparent in the NMS model than in the first-order correlated factor model.

**Fig 3:**
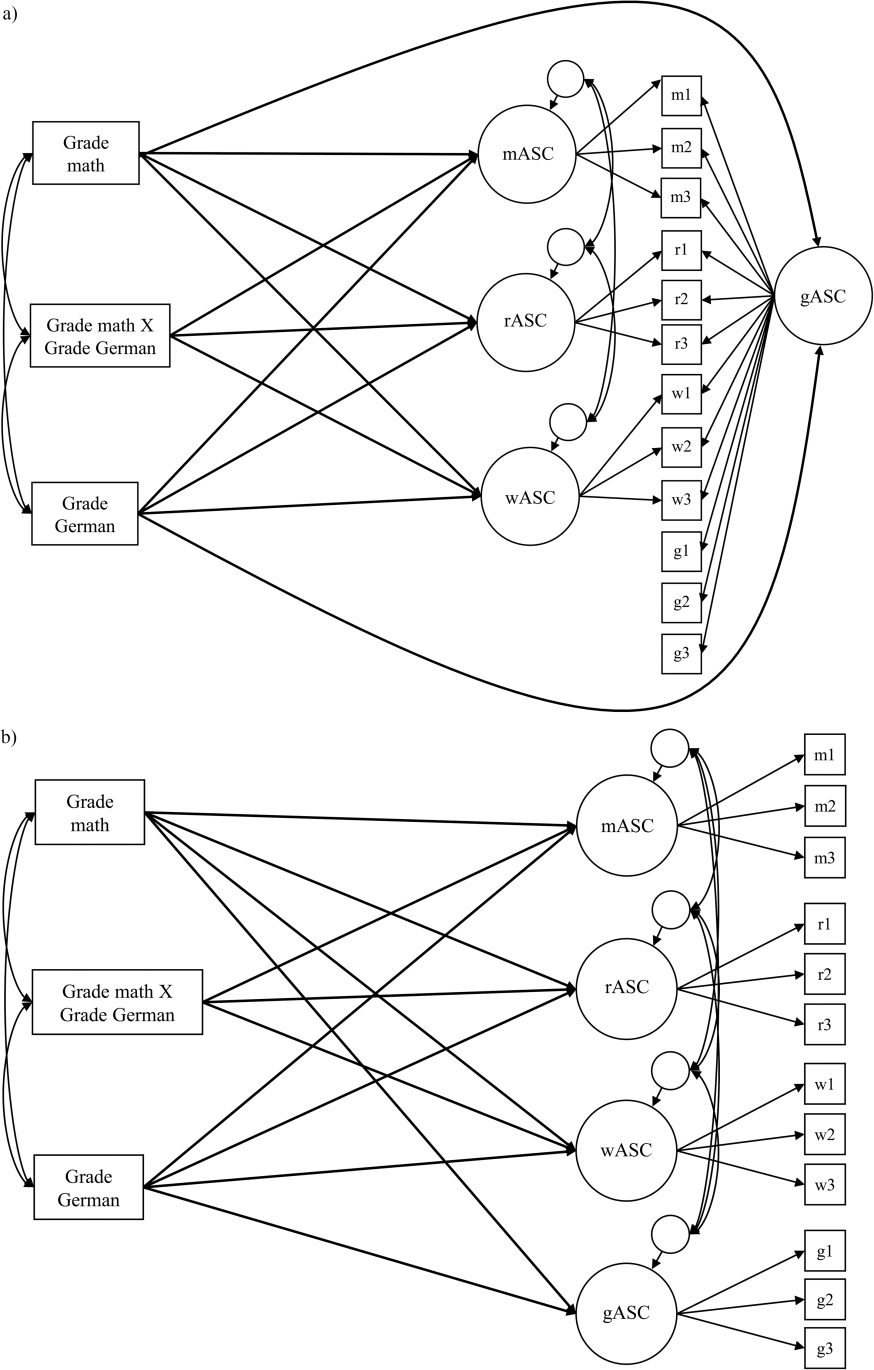
Extended I/E-Model. **(a) I/E-NMS model.** gASC = general ASC, mASC = math ASC as specific factor, wASC = writing ASC as specific factor, rASC = reading ASC as specific factor. **(b) I/E-FO model.** gASC = general ASC, mASC = math ASC, wASC = writing ASC, rASC = reading ASC as first-order factor. For the sake of clarity, residual terms of the manifest variables were omitted.

Third, to learn more about the processes underlying profile formation of ASC, we tested the I/E model within the NMS model and the first-order correlated factor model using math and German grades as achievement indicators. Fig 3(A) shows the I/E model within the NMS model; Fig 3(B) illustrates the I/E model within the first-order correlated factor model. In grades 3 and 4, we extended the I/E model by including an interaction term (math grade × German grade) as a predictor of math ASC, reading ASC, and writing ASC to test our assumption that effects of dimensional comparisons become stronger when achievements in cross-domains differ [18–19]. We expected a comparable size of the effect in grade 3 and grade 4 because the moderating effect of achievement differences should depend on children’s ability to evaluate the size of the difference between their grades in math and the native language. Research indicates that children are already able to do so at the beginning of elementary school (from age 6 onwards; e.g., [42]). To the best of our knowledge, our study is the first to extend the I/E model by an interaction term capturing the impact of grade differences in elementary school students.

## Method

### Sample and procedure

The sample is composed of a quasi-representative cross-sectional sample of German elementary school students (the norming sample of the intelligence test “THINK1–4”; [43]). The data used in this study were collected in five German federal states (Hesse, Lower Saxony, Mecklenburg-West Pomerania, North Rhine-Westphalia, and Rhineland-Palatinate) between September 2012 and January 2014. Thus, we took care to cover a broad range of federal states because of the north-south as well as east-west disparity in Germany; also, we ensured that rural and urban areas were adequately covered. Permissions for the different federal states were obtained mainly through contacts with the network of the editors of the series “Hogrefe Schultests”. The total sample comprised *N* = 3,779 students (50.2% female). The grade 1 sample consisted of 427 students (47.3% female) from 34 classes, the grade 2 sample consisted of 718 students (50.8% female) from 53 classes, the grade 3 sample consisted of 1,288 students (52.6%) from 82 classes, and the grade 4 sample consisted of 1,346 students (49.5% female) from 90 classes. Students’ mean age was 6.43 years (*SD* = .51) in grade 1, 7.63 years (*SD* = .57) in grade 2, 8.67 years (*SD* = .60) in grade 3, and 9.66 years (*SD* = .59) in grade 4. The larger samples in grades 3 and 4 were due to oversampling by design (the project THINK further addressed the transition to secondary school). In all grade levels, ASCs were assessed by self-report. Student questionnaires were administered in class.

The study was approved by the responsible authorities of the federal states of Germany. This permission required the approval of the data protection commissioners of each respective federal state. In Hesse, the study was approved on March 7, 2013 by the Hessian Ministry of Education, No.: 660.003.000–00544. In Lower Saxony, the study was approved on August 16, 2012 by the Lower Saxony State Board of Education, No.: BS 1 R.21-81402/1-26/12. In Mecklenburg-West Pomerania, the study was approved in August 2012 by the Ministry of Education, Science and Culture of Mecklenburg-West Pomerania, No.: VII 201 g. In Rhineland-Palatinate, the study was approved on May 4, 2012 by the Supervision and Service Administration of Rhineland-Palatinate, No.: 51 111-32/70-12. In North Rhine-Westphalia, permissions were given orally by the school principals who have the decision-making power in this federal state of Germany.

Student participation was anonymous and voluntary. Prior written parental consent was obtained for all participating children. Anonymity was ensured by predefined codes that were assigned by teachers, according to coding instructions. Only teachers knew which code referred to which child. The same code was assigned to each set of questionnaires completed by one family (i.e., child-parent dyads). On average, 68% of all students in each class participated in the study. The overall consent rate was 79%. Dropout was due to missing parental approval or sickness on the day of the examination. Data collection took place in all four quarters of the school year. Within each school, data were collected in the same quarter, but quarters varied between schools. There was no significant correlation between the quarters and the ASC scale means, with the exception of writing ASC in grade 3 (*r* = -.13, *p* = .016).

### Variables and measures

#### Academic self-concepts

To assess general ASC and writing, reading, and math ASC, we applied 3-item scales for each conception from the short version of the FEESS 1–2 & 3–4 [44–45]. The items were: “I am good at reading/writing/math/in school” (r1, w1, m1, g1), “I do well in reading/writing/math/school” (r2, w2, m2, g2), and “In reading/writing/math/school, I do most things well” (r3, w3, m3, g3). To facilitate responses for the first and second graders in the sample and to make ratings comparable across grade levels (the original FEESS uses a dichotomous scale in grades 1 and 2 and a four-point rating scale in grades 3 and 4), a three-point rating scale was employed with frowning, neutral, and smiling faces to indicate disagreement, indifference, and agreement. A smiley scale is easy to understand and suitable for the assessment with elementary school children (see [46]). For all students, a sample task was used to explain the smiley scale. Taking into account that not all students were yet able to read fluently in grades 1 and 2, all items were read aloud to these students.

#### School grades

For participants in grades 3 and 4, parents reported their children’s teacher-assigned school grades at the time of data collection (i.e., from their most recent report card), while no grades were reported for 1^st^ and 2^nd^ graders because children are not graded in Germany until grade 3. All parent reports were anonymous due to the teacher-assigned codes; parent questionnaires were returned in sealed envelopes, and teachers did not have access to the returned data. The instructions requested parents to provide valid information. Thus, we trusted the validity of the parent-reported grades. For the present analyses, the usual German grading scale (from 1 = *very good* to 6 = *insufficient*) was inverted so that higher numbers indicate higher achievement.

### Data analyses

First, we conducted confirmatory factor analyses (CFA) to investigate the different structural models of ASC. All model parameters were analyzed with the statistical software M*plus* 7.31 [47]. As is usually the case in educational research, students were nested within classes, which may result in distorted significance tests. Hence, we used the COMPLEX option in M*plus* to obtain correct standard errors of parameter estimates and test statistics. To account for the ordered categorical nature of the data (three-point rating scales), we conducted CFAs for ordered categorical data using the mean- and variance-adjusted weighted least square estimator (WLSMV) [48] with theta parameterization.

To evaluate model fit of the CFAs, we used the chi-square (χ2) goodness-of-fit statistic. Because this statistic is sensitive to sample size, we also used the following recommended descriptive measures of model fit [49]: (1) the root mean square error of approximation (RMSEA), which should be below .06, (2) the comparative fit index (CFI), which should exceed .95, and (3) the weighted root mean residual (WRMR), which should be below 1 [50].

We compared the NMS model with a gASC and reading ASC, writing ASC, and math ASC as correlated specific ASC factors nested under gASC (Model 1: NMS_3sf; sf for “specific factors”) against alternative structural models: an NMS model with math ASC and one comprehensive verbal ASC as specific ASC factors (Model 2: NMS_2sf); a first-order correlated factor model with general ASC, math ASC, reading ASC, and writing ASC as distinct but correlated factors (Model 3: FOCF_4f; f for “factors”), a first-order correlated factor model with general ASC, math ASC, and only one verbal ASC factor (Model 4: FOCF_3f), and a *g*-factor model in which all items were explained by a single first-order factor (Model 5: *g*-factor) (see Fig 2). Latent variables in all structural models of ASC were identified by fixing the first item loading of each factor to 1. Further, covariances between general ASC and the two or three subject-specific ASC factors were constrained to 0 in the NMS_3sf model and the NMS_2sf model. This implies that subject-specific ASC factors are truly residualized while the variance attributed to gASC is controlled for [9]. All other model parameters were freely estimated. The models were tested separately for grades 1 to 4.

Second, we tested the NMS_3sf and FOCF_4f models (i.e., Models 1 and 3) for scalar measurement invariance across grade levels through multigroup comparison. This is the best-practice approach to group comparisons regarding the structural models of ASC and a prerequisite for further invariance testing of correlations and factor means of ASCs [51]. The measurement invariance testing procedure consisted of comparing nested models differing in number of parameters, which were restricted to be invariant across groups (see, e.g., [51]). In the configural model, the same structural model was estimated simultaneously for each group, but the estimated parameters (factor loadings and thresholds) were allowed to vary between groups. In the scalar model, factor loadings for identical items and their corresponding thresholds were held equal across grades [51]. To assess scalar invariance, we used the χ^2^ difference test between the configural and the scalar model using the DIFFTEST option in M*plus*. It should be noted that with ordered categorical indicators and indicators loading on two factors, it is not possible to test the invariance of the factor loadings (i.e., metric measurement invariance) separately from scalar measurement invariance (see, e.g., [52]).

Third, under the assumption of scalar measurement invariance, we tested the hypothesis that ASC profiles are more distinct in higher grades by comparing all pairwise correlations among math ASC, reading ASC, and writing ASC between the 4 grade levels using the Wald χ^2^ test. To interpret the magnitude of correlations, we used the classification proposed by Cohen [53], with *r*s of .10, .30, and .50 indicating small, medium, and large effects, respectively.

Fourth, to investigate the assumptions of the extended I/E model, we conducted separate path analyses in grades 3 and 4. Math and German grades were included into the model as manifest variables. To avoid multicollinearity, we standardized grades beforehand within each school year and computed the interaction term between the grades in math and German. To obtain fully standardized path coefficients in the I/E model, we defined the metric in the NMS_3sf and FOCF_4f models by fixing the variance of the latent factors to 1. Additionally, to account for general achievement (equivalently to general ASC), we added paths leading from grades in German and math to the general ASC factor. Fig 3(A) shows the extended I/E model with the NMS_3sf model of ASC (I/E-NMS model). Fig 3(B) shows the extended I/E model with the FOCF_4f model of ASC (I/E-FO model).

## Results

### Descriptive statistics

Table 1 shows the means, standard deviations, and manifest correlations of all measures by grade level. In line with previous findings, descriptive statistics indicated lower mean levels of reading ASC, writing ASC, math ASC, and general ASC with increasing grade level (except for reading ASC between grades 1 and 2). Furthermore, at a descriptive level, manifest correlations between writing/reading ASC and math ASC decreased with increasing grade level; the correlation between writing and reading ASC was lower in grade 2 than in grade 1, and higher in grade 4 than in grade 3. Scale reliabilities in terms of Cronbach’s alpha (α) were acceptable to good, ranging from α = .68 to .87 across grade levels.

**Table 1:** Means, standard deviations, and manifest correlations of measures by grade level.

**Grade 1 (above diagonal) and Grade 2 (below diagonal)**										
	ASC general	ASC math	ASC writing	ASC reading			*n*	*M*	*SD*	α
ASC general	—	.52**	.42*	.41***			422	1.73	.41	.68
ASC math	.51**	—	.44**	.38***			365	1.75	.44	.83
ASC writing	.44**	.23**	—	.53**			367	1.75	.42	.77
ASC reading	.40**	.22**	.52**	—			363	1.73	.47	.81
*n*	716	356	356	370						
*M*	1.69	1.69	1.69	1.76						
*SD*	.40	.46	.46	.42						
α	.74	.83	.81	.77						
**Grade 3 (above diagonal) and Grade 4 (below diagonal)**										
	ASC general	ASC math	ASC writing	ASC reading	Grade math	Grade German	*n*	*M*	*SD*	α
ASC general	—	.52**	.44*	.42***	.35**	.31**	726	1.60	.46	.74
ASC math	.53**	—	.17**	.16***	.37**	.04	334	1.60	.51	.86
ASC writing	.43**	.15**	—	.35**	.14*	.29**	333	1.54	.51	.82
ASC reading	.40**	.13**	.40**	—	.20**	.31**	338	1.70	.44	.82
Grade math	.46**	.54**	.10**	.14**	—	.65**	1,174	5.0	.76	
Grade German	.49**	.23**	.38**	.38**	.67**	—	1,182	4.9	.74	
*n*	1,255	854	862	370	1,173	1,169				
*M*	1.56	1.58	1.46	1.67	4.82	4.69				
*SD*	.44	.49	.54	.44	.88	.85				
α	.74	.86	.87	.87						

Descriptive statistics were computed using IBM SPSS Statistics version 22.0 (SPSS Inc., USA). Scale reliability was calculated using Cronbach’s α. † p < .10

* p < .05

** p < .01

*** p < .001.

### Fit of the different structural models of ASC in grades 1 to 4

Table 2 shows the model fit results of the five different structural models of ASC by grade level. As expected, the NMS_3sf and the FOCF_4f models fitted the data well. In both models, latent constructs were measured adequately by their respective indicators at all grade levels (standardized factor loadings: NMS_3sf > .47, all *p*s < .001; FOCF_4f model > .68, all *p*s < .001; see Table A1 in S1 Tables). The models assuming only one verbal ASC factor (NMS_2sf and FOCF_3f) showed worse fit than the models distinguishing reading ASC and writing ASC. The *g*-factor model of ASC showed insufficient fit.

**Table 2:** Model fit results of the five structural models (M1: NMS_3sf; M2: NMS_2sf; M3: FOCF_4f; M4: FOCF_3f; M5: g-factor) of academic self-concept by grade level.

Model	*N*	χ^2^	*df*	*p*	CFI	RMSEA [90% CI]	WRMR
Grade 1							
M1: NMS_3sf	427	45.811	42	.317	.999	.015 [.000;.037]	.477
M2: NMS_2sf		100.129	44	< .001	.980	.055 [.041;.069]	.860
M3: FOCF_4f		73.069	48	.011	.991	.035 [.017;.050]	.701
M4: FOCF_3f		133.571	51	< .001	.970	.062 [.049;.074]	1.113
M5: *g*-factor		235.277	54	< .001	.935	.089 [.077;.100]	1.791
Grade 2							
M1: NMS_3sf	718	45.598	42	.325	.999	.011 [.000;.028]	.491
M2: NMS_2sf		136.243	44	< .001	.975	.054 [.044;.064]	1.109
M3: FOCF_4f		78.234	48	.004	.992	.030 [.017;.041]	.776
M4: FOCF_3f		148.701	51	< .001	.973	.052 [.042;.061]	1.330
M5: *g*-factor		410.464	54	< .001	.902	.096 [.087;.105]	2.745
Grade 3							
M1: NMS_3sf	726	58.999	42	.043	.997	.024 [.005;.037]	.590
M2: NMS_2sf		136.243	44	< .001	.975	.054 [.044;.064]	1.109
M3: FOCF_4f		112.500	48	< .001	.987	.043 [.033;.053]	1.073
M4: FOCF_3f		245.766	51	< .001	.961	.073 [.064;.082]	1.944
M5: *g*-factor		851.055	54	< .001	.839	.143 [.134;.151]	4.057
Grade 4							
M1: NMS_3sf	1259	143.424	42	< .001	.996	.044 [.036;.052]	.890
M2: NMS_2sf		965.181	44	< .001	.962	.129 [.122;.136]	3.100
M3: FOCF_4f		239.363	48	< .001	.992	.056 [.049;.063]	1.302
M4: FOCF_3f		818.168	51	< .001	.968	.109 [.103;.116]	3.238
M5: *g*-factor		2340.188	54	< .001	.905	.183 [.177;.190]	6.718

CFI = comparative fit index; RMSEA = root mean square error of approximation; WRMR = weighted root mean square residual. M1: NMS_3sf = Nested Marsh/Shavelson model of ASC with math ASC, writing ASC, and reading ASC as correlated specific factors; M2: NMS_2sf = Nested Marsh/Shavelson model of ASC with math ASC and verbal ASC as correlated specific factors; M3: FOCF_4f = First-order correlated factor model with general ASC, math ASC, writing ASC, and reading ASC as factors; M4: FOCF_3f = First-order correlated factor model with general ASC, math ASC and verbal ASC as factors; M5: g-factor = First-order factor model in which all ASC items load on one single factor.

With reference to McDonald ([54]; see also [55]), we used omega (ω) as the reliability coefficient for NMS_3sf and FOCF_4f, the two structural models of ASC favored by our results. ω can be interpreted as a measure of internal consistency within a latent variable approach. Scale reliability was excellent, ranging from .81 to .96 for both models (see Table A2 in S1 Tables).

### Measurement invariance across grade levels

Table 3 shows the results of the stepwise investigation of measurement invariance across grade levels of the NMS_3sf and the FOCF_4f models. Importantly, the nonsignificant χ^2^ difference test between the configural and the scalar invariant models indicated strong factorial measurement invariance for both the NMS_3sf and the FOCF_4f model, allowing us to compare correlations of factors as well as mean differences in factor scores across grade levels [56]. The procedure and the results of latent mean level differences tests in factor scores across grade levels are presented in S1 Appendix.

**Table 3:** Results of the measurement invariance tests for the NMS model (M1: NMS_3sf) and the first-order correlated factor model (M3: FOCF_4f), both with math ASC, writing ASC, and reading ASC as distinct factors.

Model	Invariance level	χ^2^	*df*	Δχ^2^ *(*Δ*df)*	*p*	CFI	RMSEA [90% CI]	WRMR
M1	configural	268.741	168		< .001	.997	.028 [.021;.034]	1.266
	scalar	325.141	243		< .001	.998	.021 [.014;.026]	1.556
	configural vs. scalar			87.958 (75)	.145			
M3	configural	463.313	192		< .001	.992	.042 [.038;.047]	1.983
	scalar	494.622	240		< .001	.992	.037 [.037;.032]	2.060
	configural vs. scalar			52.268 (48)	.312			

CFI = comparative fit index; RMSEA = root mean square error of approximation; CI = confidence interval; WRMR = weighted root mean square residual. M1: NMS_3sf = Nested Marsh/Shavelson model of ASC with math ASC, writing ASC, and reading ASC as correlated specific factors; M3: FOCF_4f = First-order correlated factor model with general ASC, math ASC, writing ASC, and reading ASC as factors.

### Profile formation of academic self-concept

**Fig 4:**
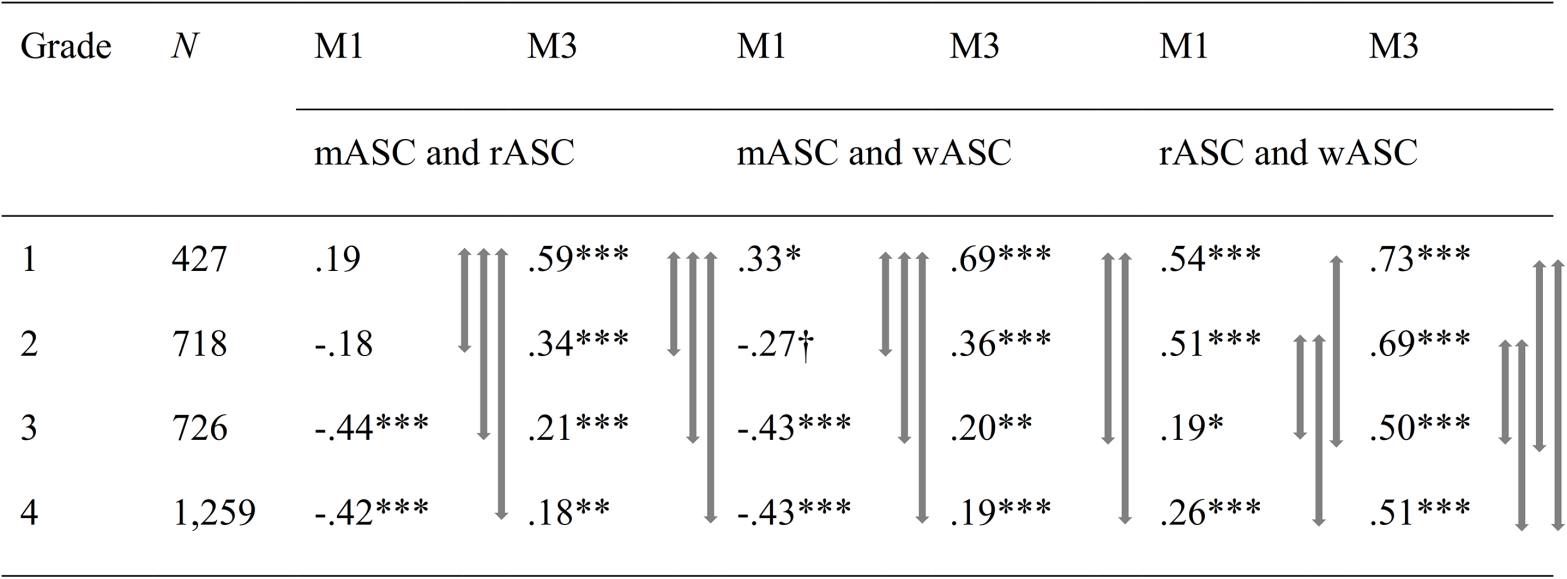
Latent correlations between academic self-concepts in math, writing, and reading by model (M1: NMS_3sf; M3: FOCF_4f) and grade level. Despite equal nomenclature, mASC = math ASC, wASC = writing ASC, and rASC = reading ASC are first-order correlated factors in the FOCF_4f model and specific factors that are residualized by gASC in the NMS_3sf model; thus, factors from both models are not directly comparable. M1: NMS_3sf = Nested Marsh/Shavelson model of ASC with math ASC, writing ASC, and reading ASC as correlated specific factors; M3: FOCF_4f = First-order correlated factor model with general ASC, math ASC, writing ASC, and reading ASC as factors.† *p* < .10 * *p* < .05 ** *p* < .01 *** *p* < .001. Gray arrows mark significant differences between correlation coefficients of different grades. How to read the table (e.g., column 4, M1, mASC and rASC): The difference in the correlation between mASC and rASC in Model 1 was significant between grades 1 and 2, 1 and 3, and 1 and 4.

For the NMS_3sf and the FOCF_4f models, descriptive results revealed lower correlations in higher grades among ASCs of noncorresponding domains (math and reading/writing) and within the verbal domain (reading and writing) (see Fig 4). Both sizes and signs of correlations differed between the two models. In the NMS_3sf model, reading ASC and math ASC were weakly and positively correlated in grade 1; their correlation was weak and negative in grade 2, and even more negative and of medium size in grade 3, but then remained at about the same level in grade 4. The correlation of writing ASC and math ASC showed the same pattern, except that the positive correlation was larger, that is, of medium size in grade 1. In contrast, in the FOCF_4f model, correlations between reading ASC and math ASC and between writing ASC and math ASC were positive at every grade level and lower in size in higher grades. Specifically, correlations between math ASC and writing ASC/reading ASC were high and positive in grade 1, of medium size and positive in grade 2, and weak and positive in grades 3 and 4.

Regardless of the structural model investigated, correlations between writing ASC and reading ASC were always positive, and they were lower in higher grades. However, the size of the correlations was larger in the FOCF_4f model than in the NMS_3sf model.

For both models, Wald χ^2^ tests of the difference of correlations between reading ASC and math ASC were significant between grades 1 and 2, whereas the correlations between writing ASC and math ASC differed significantly between the two grades in the NMS_3sf model only. Further, in both models, significantly different correlations between reading ASC and math ASC were observed between grades 1 and 3 and between grades 1 and 4. In both models, the correlation between writing ASC and math ASC differed significantly between grades 1 and 3 and between grades 1 and 4, but not between grades 2 and 3, 2 and 4, or 3 and 4 (see Fig 4).

Contrary to our expectations, which were derived from the differential distinctiveness hypothesis, both models showed significantly lower correlations between writing ASC and reading ASC in grade 3 compared to grades 2 and 1. However, correlations between writing ASC and reading ASC were slightly higher in grade 4 than in grade 3, but only on a descriptive level.

In sum, in both models the pattern of results indicated that ASC profiles were formed early, that is, within the first three years of school. Profile formation for math ASC and verbal (i.e., reading) ASC occurred within the first two years of school, whereas results for the profile formation within the native language (i.e., reading ASC and writing ASC) suggested a time frame of the first three years. However, results concerning profile formation between math ASC and writing ASC differed between the two models. Results of the NMS_3sf model suggested profile formation to take place within the first two years of school, whereas results of the FOCF_4f model suggested a time frame of the first three years.

### Test of the extended I/E model

We tested the extended I/E-model within the NMS model (Model I/E-NMS) and within the first-order correlated factor model (Model I/E-FO) in grades 3 and 4 separately. Both models fitted the data well, though fit was slightly better for the I/E-NMS model than for the I/E-FO model (see Table 4).

**Table 4:** Model fit results of the extended I/E-model within the NMS model of ASC (Model I/E-NMS) and within the first-order correlated factor model of ASC (Model I/E-FO) for grades 3 and 4.

Model	*N*	χ^2^	*df*	*p*	CFI	RMSEA [90% CI]	WRMR
Grade 3							
I/E-NMS	620	122.791	67	< .001	.981	.037 [.026;.047]	0.854
I/E-FO		158.859	73	< .001	.971	.044 [.034;.053]	1.093
Grade 4							
I/E-NMS	1079	294.426	67	< .001	.978	.050 [.044;.057]	1.158
I/E-FO		242.453	73	< .001	.982	.053 [.047;.059]	1.310

CFI = comparative fit index; RMSEA = root mean square error of approximation; WRMR = weighted root mean square residual.

#### External comparisons

In both grades and in both models, paths from school grades to the specific ASC in the corresponding domain were significant and positive, indicating significant effects of external comparisons (see Table 5).

**Table 5:** Standardized path coefficients for the extended I/E model within the NMS model of ASC (I/E-NMS Model) and within the first-order correlated factor model of ASC (I/E-FO Model) in grades 3 and 4 and variance explained by grades (R^2^) for math ASC, writing ASC, reading ASC, and general ASC.

Grade	3		4	
Model	I/E-NMS Model	I/E-FO Model	I/E-NMS Model	I/E-FO Model
Path	β	β	β	β
External Comparisons				
Grade Math → gASC	.44***	.44***	.44***	.45***
Grade German →gASC	.28**	.28**	.58***	.59***
Grade Math → mASC	.78***	.84***	.86***	.92***
Grade German → wASC	.37***	.48***	.48***	.72***
Grade German → rASC	.42***	.50***	.49***	.71***
Dimensional Comparisons				
Grade Math → wASC	-.40**	-.05	-.56***	-.23**
Grade Math → rASC	-.22**	.09	-.43***	-.16*
Grade German → mASC	-.86***	-.34**	-.92***	-.22**
Interaction Grade math and Grade German				
Grade Math x German → mASC	.40**	.25**	.13***	.10**
Grade Math x German → wASC	.18*	.14*	.16***	.14***
Grade Math x German → rASC	.20**	.15**	.14***	.14***
*R*^2^				
gASC	.29***	.29***	.46***	.46***
mASC	.41***	.29***	.35***	.36***
wASC	.13*	.15**	.19***	.24***
rASC	.11**	.21***	.14***	.25***

Despite equal nomenclature, mASC = math ASC, wASC = writing ASC, and rASC = reading ASC are first-order correlated factors in the I/E-FO model and specific factors that are residualized by gASC in the I/E-NMS model; thus, factors from the two models are not directly comparable.

* p < .05

** p < .01

*** p < .001.

#### Dimensional comparisons

In the I/E-NMS model, paths from school grades to the specific ASC in the noncorresponding domain were negative and statistically significant, suggesting contrast effects of dimensional comparisons in both grades (Table 5). In the I/E-FO model, all paths reflecting dimensional comparison processes were negative and significant, except for the paths leading from the math grade to the noncorresponding ASCs in reading and writing in Grade 3.

The comparison of both models revealed that paths reflecting external comparisons were slightly larger in the I/E-FO model, whereas paths reflecting dimensional comparisons were larger in the I/E-NMS model. Moreover, in grade 3, the paths reflecting dimensional comparisons from the math grade to the noncorresponding ASCs (reading and writing) did not reach significance in the I/E-FO model.

#### Achievement differences: Interaction with school grades

**Fig 5:**
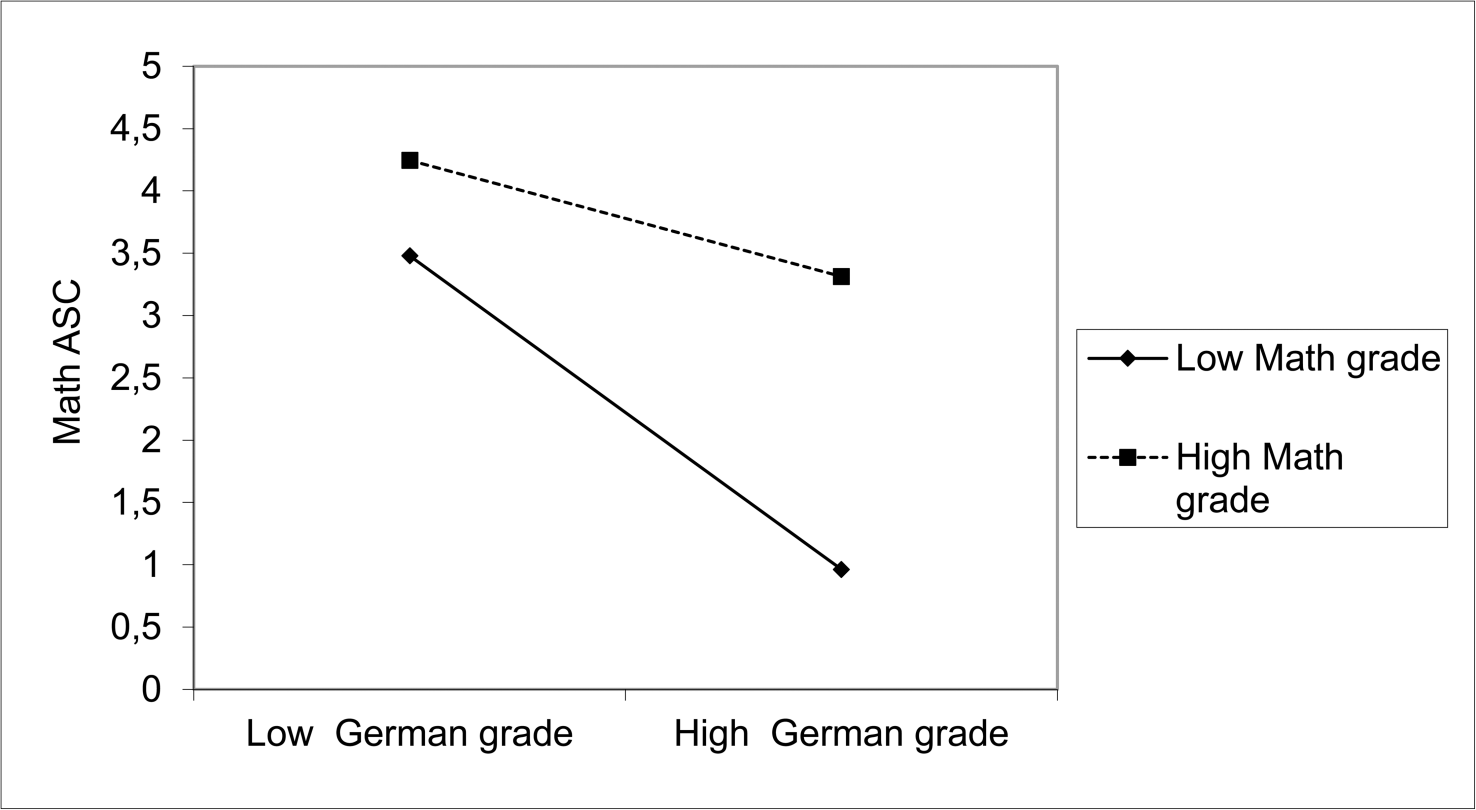
Example: Effects of the interaction term (math × German grade) on academic self-concept in math in grade 3. The intercept is hypothetical because it cannot be estimated using WLSMV.

In both the I/E-NMS model and the I/E-FO model, the interaction term between school grades in German and math significantly predicted math ASC, reading ASC, and writing ASC (Table 5). The effect of the interaction term was slightly higher in the I/E-NMS model compared to the I/E-FO model. For both models, the size of the interaction effect was comparable in grade 3 and grade 4. This was demonstrated by a nonsignificant χ^2^ difference test between a model with equality constraints and a model without equality constraints of the effects of the interaction between grades 3 and 4 (I/E-NMS model: Δχ^2^ (3) = 5.002, *p* = .172; I/E-FO model: Δχ^2^ (3) = 4.773, *p* = .189). Fig 5 provides an example of the interaction effect between school grades in German and math for math ASC within the I/E-NMS model in grade 3.

## Discussion

The aim of the present study was to investigate an important aspect of differentiation of ASC: How do profiles of students’ strengths and weaknesses form across the elementary school years, and which factors influence profile formation? A key characteristic of our study was that we juxtaposed the NMS model, which provides a very clear picture of students’ ASC profiles, with other structural models of ASC, specifically the often-used first-order correlated factor model. To this end, we drew on data from a quasi-representative sample of German elementary school students spanning grades 1 to 4.

Our first step was to model different structural assumptions of ASC. Whereas the multidimensional nature of ASC is well documented in the literature, it is still a matter of debate how one should account for its hierarchical nature, specifically for the assumption of a general ASC at the apex. By testing different structural models of ASC, our study added to existing knowledge. First, we are the first to demonstrate that the NMS model accounts for both a general ASC and for the multidimensionality of ASC already in elementary school students. Second, for this group of students we showed that verbal ASC can be meaningfully differentiated into reading ASC and writing ASC. We found profile formation to take place early during the first three years of elementary school, indicated by initially positive correlations between math and native language (i.e., reading and writing) ASCs decreasing toward zero (in the first-order correlated factor model) or by changing from positive to negative (in the NMS model). Further, results indicated a profile formation within the native language by the decreasing positive correlations between reading ASC and writing ASC during the first three years of school.

Within the I/E model, we then tested whether dimensional comparisons influence profile formation in grades 3 and 4. Overall, our findings supported the assumptions of the I/E model. However, effect sizes differed slightly depending on the structural model used. Expanding the I/E model by an interaction term (math grade × German grade), we showed that achievement differences moderated the effect of dimensional comparisons. With increasing differences between students’ grades in math and German, contrast effects of dimensional comparisons became stronger.

### Limitations

Our study analyzed cross-sectional data only (i.e., samples of students at different grade levels). However, such data can be quite useful for demonstrating age or grade differences and interindividual differences in developmental processes [57]. Further, cross-sectional data have proven fruitful for investigating research questions pertaining to development in general (e.g., [34, 58]). Considering that only longitudinal data can provide information about age- (or grade-) related changes or interindividual differences in developmental trajectories (e.g., [59]), our findings cannot be interpreted in terms of actual developmental *processes* per se.

Hence, replicating our findings longitudinally is a promising avenue of future research. Our present focus, however, was on profile formation. Therefore, we investigated dimensional comparisons as a source of information for the different subject-specific ASCs as well as external comparisons when testing the I/E model. However, the formation of subject-specific ASC is influenced by further sources and additional frames of references [12], for example, intraindividual comparisons over time. It is still unclear how these comparisons impact ASC profile formation and how they interact with each other (see [14]). Hence, for understanding ASC profile formation comprehensively, future research should also consider these factors.

The fact that only one achievement indicator was available to predict writing ASC and reading ASC in our test of the I/E model is one further limitation which resulted in a mismatch of predictor (grades) and criterion (ASC) levels of generality. In line with the specificity matching principle [60], specific constructs should be used to predict specific outcomes, whereas general constructs should be used to predict general outcomes [10]. Thus, future studies should consider using separate achievement indicators for reading and writing. This would also allow clarifying whether there is (1) an assimilation effect of dimensional comparison processes between reading and writing achievement and their noncorresponding ASC within the verbal domain or (2) a contrast effect between reading/writing achievement and math ASC. Utilizing separate achievement indicators for reading and writing, Ehm et al. [16] confirmed the I/E model for reading ASC and math ASC in grade 3 but found no contrast effect between either math achievement and writing ASC or writing achievement and math ASC. However, they observed assimilation effects of dimensional comparison processes between writing ASC and reading ASC. It should be noted, however, that Ehm et al.’s [16] study cannot be compared directly to ours because they used standardized achievement test scores, and these could influence the results of the I/E model (see [15]). Previous research on the I/E model indicates that the operationalization of achievement (grades vs. standardized achievement test scores) moderates the effects of external and dimensional comparison processes. Correlations between math and verbal achievement were substantially larger for standardized test scores, whereas the different ASCs were more strongly correlated with achievement in the corresponding domain when grades were used (meta-analysis of the I/E model, [15]; see also [40]).

### Structure and profile formation of academic self-concept in elementary school students

At all grade levels, the NMS model with general ASC at the apex and reading ASC, writing ASC, and math ASC as specific factors nested under general ASC provided a very good fit to the data. That is, the theoretical assumptions that (1) ASC is multidimensional (Marsh/Shavelson model) and that (2) general ASC is the central construct in ASC (Shavelson model), which are incorporated in the NMS model, could be supported for elementary school children, too. A special feature of this model is that the correlations among specific factors (i.e., math ASC, writing ASC, and reading ASC) depict students’ ASC profiles, that is, how students subjectively assess their strengths and weaknesses in different school subjects independent of their general level of ASC. From a theoretical stance, this model is plausible: When students compare their perceived ability in various subjects, the general level of ASC should be unimportant for evaluating one’s profile of strengths and weaknesses. In contrast to the NMS model, subject-specific ASC factors still contain variability attributable to a general ASC in the first-order correlated factor model of ASC, which makes it more difficult to interpret correlations between subject-specific ASCs in terms of students’ self-concept profiles. Hence, we think that the NMS model may be better suited for investigating profile formation of ASC than a first-order correlated factor model of ASC, although the latter demonstrated good fit to our data.

Using the NMS model, we identified positive correlations between math and reading/writing ASC in grades 1 and 2, but negative correlations in grades 3 and 4, which is in line with our expectations. This finding suggests that (1) the ASC profile developed with increasing grade level and that (2) profile formation started early. Comparing these results with those for the first-order correlated factor model, where correlations between math and writing/reading ASC were positive at every grade level (but lower in the higher grades as well), changes became more apparent in the NMS model. Findings for the NMS model revealed a shift from positive to negative correlations between math and writing/reading ASC and increasingly negative correlations between these different facets of ASC with increasing grade level. In sum, in line with the differential distinctiveness hypothesis, our findings support the assumption that math ASC and native language ASC become more differentiated with age [34].

In Germany, where our study took place, achievement feedback through explicit grading usually starts in grade 3. However, according to our results, elementary school students’ profile formation begins in grade 2, suggesting that explicit grading may not be a necessary condition for profile formation of ASC. One possible reason is that grades are only one source of achievement-related feedback. External feedback from significant others (teachers, parents, peers) may also reinforce profile formation of ASC (see, e.g., [32]). More specifically, several studies showed that children increasingly use available information like ability feedback from significant others to evaluate their own ability during elementary school (see [31, 61] for a summary).

Drawing on the differential distinctiveness hypothesis [34], we further assumed an integration of the different skill-specific ASCs (i.e., writing and reading) indicated by stable or increasing positive correlations with grade level. It should be noted that the differential distinctiveness hypothesis has not yet been explicitly tested with regard to different skill-specific ASCs.

In contrast to expectations, the correlation between writing ASC and reading ASC was significantly lower in grade 3 as compared to grade 2, and only slightly higher (and nonsignificant) again in grade 4 as compared to grade 3. This correlational pattern indicated increasing differentiation within the verbal ASC profile during the first three years of school. How can this be explained? One possibility is that the assumption of the differential distinctiveness hypothesis (i.e., that self-concepts associated with the same higher-order factor are more closely associated) does not apply to the differentiation of skill-specific ASC. The original differential distinctiveness hypothesis rests on the higher-order factor constellation within the structural model of the entire self-concept (Marsh/Shavelson model). However, this model does not include the different skill-specific ASCs of each subject-specific ASC.

Further, we hypothesized stronger integration of children’s different skill-specific ASCs, drawing on results from previous studies that showed high correlations between skill-specific ASCs of (native) language ASC for different age groups [35–37]. However, all of these studies (and our study, too) relied on cross-sectional data, which hampers the interpretation of results as a developmental process. Hence, it is also possible that skill-specific ASC becomes more differentiated with age, but not to the same extent as subject-specific ASC, for example, math ASC and verbal ASC. This seems plausible because students might learn that writing and reading are different skills—for instance, a student might be better at writing essays but might not understand texts very well when reading them. However, it is unlikely that a student could write a good essay without being able to understand a text. Therefore, the correlations between writing ASC and reading ASC might be substantial but not perfect, as indicated by previous studies as well as our study. To conclude, future studies using longitudinal data are needed to clarify differentiation within (native) language ASC.

### The extended I/E model and factors moderating dimensional comparisons

Although profile formation was observed even without explicit grading (in grades 1 and 2), our findings for the extended I/E model suggest that grading contributes to this process. For both ASC models, most assumptions of the extended I/E model could be replicated in grades 3 and 4 using teacher-assigned grades and the interaction between them as achievement indicators. All paths representing external comparison processes were positive and significant in both models. Findings for internal (dimensional) comparison processes partly depended on the specific ASC model used. In grade 4, all paths representing internal comparison processes were negative and significant regardless of the model, whereas in grade 3 this was only the case for the NMS model. When the I/E model was investigated with the first-order correlated factor model, the paths leading from math grade to reading ASC and writing ASC failed to reach significance in grade 3. One possible explanation for this discrepancy is the specific feature of the NMS model: Subject-specific ASC factors are residualized from general ASC. In the NMS model, correlations between subject-specific ASC in math and reading/writing were moderately negative, reflecting the finding that students see themselves as either “math” or “verbal” persons ([10]; see also [7]).

Statistically, moderately negative correlations between the different subject-specific ASCs should result in larger negative paths between noncorresponding grades and ASC than when correlations are small but positive, which was the case for the I/E-first-order correlated factor model. When comparing the two I/E models, paths indicating external comparison processes revealed slightly higher coefficients in the first-order correlated factor model than in the NMS model, while path coefficients for internal comparison processes were slightly higher (and negative) in the NMS model. An explanation may be that, again, subject-specific ASC in math, reading, and writing are residualized from general ASC in the I/E-NMS model. We found teacher-assigned grades in math and German to be related to both specific ASC and general ASC. Residualization therefore results in smaller positive path coefficients for external comparisons in the I/E-NMS model [10]. In the I/E-first-order correlated factor model, the effects of internal comparisons are confounded with those of external comparisons referring to general ASC (because subject-specific ASC still contains variance attributed to the general ASC). Hence, contrast effects of internal comparisons were smaller whereas effects of external comparison were slightly stronger in the I/E-first-order correlated factor model.

Overall, our study demonstrates that the investigated effects are robust across different structural models of ASC and that the I/E model is generalizable to elementary school students—a set of findings which is well in line with previous research (see [15, 17]).

Further, differences in detail suggest that when studying the effects of internal and external comparison processes, the moderating role of the structural models of ASC should be kept in mind.

One factor that reinforces contrast effects of dimensional comparisons between subjects pertaining to different domains is the achievement difference between subjects. The higher this discrepancy, the stronger the contrast effects of dimensional comparison processes [18–19], which implies a more pronounced profile formation of ASC. However, this factor has been much neglected in I/E model research to date. Our findings suggest that the moderating effect of grade differences in cross-domains generalizes to elementary school students in grades 3 and 4. However, objective achievement differences may not be the only moderators of the effect of dimensional comparisons. Studies with secondary school students have shown, for example, that subjective beliefs, specifically, beliefs in a negative interdependence between math and verbal abilities [62] or in perceived similarity of school subjects [63], may also be an important factor. We therefore recommend that future studies of the I/E model consider objective factors such as achievement differences (e.g., as an interaction term), subjective factors such as beliefs, and their interplay as moderators of dimensional comparisons.

In general, further research is needed to test whether findings generalize across subgroups such as gender or students from different school systems.

### Conclusions and implications

In sum, the NMS model of ASC [9–11] may guide future research on the profile formation of ASC. Our findings indicate that this process starts during the first two to three years of elementary school, even without explicit feedback through teacher-assigned grades. However, grade feedback exacerbates differentiation in grades 3 and 4, especially when grades differ between contrasting domains. Our study showed that the effects of dimensional comparison processes depend both on the grade level under examination and on the chosen structural model of ASC. Both factors should, therefore, be taken into account when studying these effects.

Our findings have theoretical, methodological, as well as practical implications for the research community.

First, different structural models of ASC are suitable for different research questions. When investigating profile formation, a nested ASC model seems to be preferable over a first-order correlated factor model because this model allows controlling for the level of general ASC in students’ profiles. Thus, students’ ASC profiles of subjective strengths and weaknesses (in terms of subject-specific ASCs) as well as central aspects of the shape of these profiles (in terms of correlations among subject-specific ASCs) can be depicted. The nested ASC model, as applied in the present study, has the advantage that a general ASC factor is psychometrically well defined by the items directly measuring general ASC. Further, the general ASC factor in this model is relatively independent of the subjects investigated. Hence, the present nested ASC model might be especially well suited in cases where not all subjects that are taught in elementary school are studied, as was the case in our study (see [25, 64]; see also [10]).

Alternatively, one may also specify a so-called complete bifactor model that includes a general factor and specific factors for all subject-specific academic self-concept s as well as a specific factor that influences the items measuring general ASC. This model has the advantage that relations of covariates (e.g., gender) to the specific factor of items measuring general academic self-concept can be investigated. Such models often approximate the empirical data even better than the nested-factor model applied in the present study [65]. However, the complete bifactor representation has two disadvantages (see [64]). Compared to the nested-factor model applied in the present study, in the complete bifactor model, the general factor is psychometrically less well defined as it depends more strongly on the subjects investigated. Further, complete bifactor models often tend to show irregular loading patterns or vanishing variances of the specific factors for general measures. Such anomalous results are not well aligned with the underlying theories.

Irrespective of whether a complete bifactor model or a nested-factor model is chosen to study academic self-concepts, one should take into account that in these models, a significant share of variance is extracted from subject-specific measures, which renders the interpretation of correlations between subject-specific factors and external variables more complex. Moreover, mean levels of subject-specific ASCs are more complex to interpret in the NMS model (or the complete bifactor model) than in the first-order correlated factor model (but see [10, 66]), which makes the latter more attractive when mean level changes of subject-specific ASC are examined.

Second, from a practical point of view, ASC profiles (i.e., knowing one’s own strengths and weaknesses) are useful for self-evaluations and may thus facilitate decision making in educational settings [14]. However, this may apply only when a student’s ASC profile corresponds to actual individual strengths and weaknesses, but not when subjective factors (e.g., beliefs in a negative interdependence of math and verbal abilities; see [62]) reinforce dimensional comparisons between cross-domain subjects. Previous research has indicated that self-concept intervention approaches which focus on enhancing comprehensive and appreciative feedback on students’ performance in a particular subject were effective in promoting positive ASC ([67], see also [68]). Such interventions may also be especially suitable for promoting the development of ASC profiles that correspond to students’ actual strengths and weaknesses.

This study has demonstrated that student profiles of ASC start to develop very early in elementary school education. Armed with this knowledge, practitioners should consider implementing interventions designed to promote ASC in students right from the start of their school careers.

## Supporting information

S1 Tables Additional tables. (DOCX)

S1 Appendix Procedure and results of mean difference testing of general ASC, reading ASC, writing ASC, math ASC between grade levels. (DOCX)

S1 Data&Syntax SPSS dataset and Mplus syntax. (ZIP)
