# Supplementary material for: Profile formation of academic self-concept in elementary school students in grades 1 to 4
Source: PLoS One. 2017 May 18;12(5):e0177854. doi: 10.1371/journal.pone.0177854 (PMC5436832; doi:10.1371/journal.pone.0177854)
Supplement: S1 Tables — (DOCX) [file pone.0177854.s001.docx]

| **Table A1. Standardized Factor-Loadings for the NMS Model (M1: NMS_3sf) and the First-Order Correlated Factor Model of Academic Self-Concept (ASC) (M3: FOCF_4f), both with Math ASC, Writing ASC, and Reading ASC Factors, for Grades 1 to 4.** | | | | | | | | |
| --- | --- | --- | --- | --- | --- | --- | --- | --- |
| Grade | 1 | | 2 | | 3 | | 4 | |
| Model | M1 | M3 | M1 | M3 | M1 | M3 | M1 | M3 |
| gASC |  |  |  |  |  |  |  |  |
| a1 | .733 | .677 | .790 | .791 | .742 | .755 | .737 | .740 |
| a2 | .799 | .805 | .749 | .762 | .807 | .862 | .802 | .804 |
| a3 | .839 | .758 | .796 | .745 | .875 | .815 | .865 | .875 |
| m1 | .722 |  | .675 |  | .680 |  | .673 |  |
| m2 | .696 |  | .636 |  | .643 |  | .661 |  |
| m3 | .700 |  | .651 |  | .654 |  | .673 |  |
| r1 | .512 |  | .483 |  | .533 |  | .478 |  |
| r2 | .549 |  | .510 |  | .514 |  | .530 |  |
| r3 | .625 |  | .546 |  | .596 |  | .608 |  |
| w1 | .511 |  | .558 |  | .519 |  | .492 |  |
| w2 | .578 |  | .568 |  | .532 |  | .546 |  |
| w3 | .660 |  | .639 |  | .574 |  | .584 |  |
| mASC |  |  |  |  |  |  |  |  |
| m1 | .592 | .923 | .624 | .931 | .649 | .954 | .640 | .936 |
| m2 | .653 | .941 | .671 | .900 | .700 | .929 | .717 | .958 |
| m3 | .468 | .866 | .490 | .828 | .508 | .839 | .521 | .865 |
| rASC |  |  |  |  |  |  |  |  |
| r1 | .755 | .880 | .789 | .908 | .808 | .960 | .797 | .915 |
| r2 | .768 | .949 | .790 | .937 | .740 | .892 | .839 | .996 |
| r3 | .540 | .868 | .523 | .800 | .530 | .813 | .594 | .865 |
| wASC |  |  |  |  |  |  |  |  |
| w1 | .656 | .804 | .733 | .903 | .780 | .917 | .795 | .933 |
| w2 | .704 | .876 | .708 | .913 | .760 | .917 | .838 | .996 |
| w3 | .509 | .890 | .504 | .828 | .518 | .803 | .566 | .865 |
| All factor-loadings were significant (*p*< .001). a1/a2/a3 = Items of general ASC; m1/m2/m3 = Items of math ASC; r1/r2/r3 = Items of reading ASC; w1/w2/w3 = Items of writing ASC. Despite equal nomenclature in M1 and M3, mASC = math ASC, wASC = writing ASC and rASC = reading ASC are first-order correlated factors in the FOCF_4f model and specific factors that are residualized by gASC in the NMS_3sf model; thus, factors from both models are not directly comparable. M1: NMS_3sf = Nested Marsh/Shavelson-model of ASC with math ASC, writing ASC, and reading ASC as correlated specific factors; M3: FOCF_4f = First-order correlated factor model with general ASC, math ASC, writing ASC, and reading ASC as factors. | | | | | | | | |

| **Table A2. Reliability Coefficient Omega (ω; McDonald, 1999) for Factors of General Academic Self-Concept (ASC) and Math ASC, Writing ASC, and Reading ASC by Grade Level and Model (M1: NMS_3sf; M3: FOCF_4f).** | | | | | | | | |
| --- | --- | --- | --- | --- | --- | --- | --- | --- |
| Grade | 1 | | 2 | | 3 | | 4 | |
| Factors | M1 | M3 | M1 | M3 | M1 | M3 | M1 | M3 |
| gASC | .95 | .81 | .95 | .81 | .95 | .85 | .96 | .85 |
| mASC | .85 | .93 | .83 | .92 | .87 | .92 | .88 | .95 |
| rASC | .88 | .98 | .87 | .92 | .88 | .92 | .92 | .94 |
| wASC | .82 | .89 | .85 | .92 | .86 | .92 | .91 | .94 |
| Despite equal nomenclature in M1 and M3, mASC = math ASC, wASC = writing ASC and rASC=reading ASC are first-order correlated factors in the FOCF_4f model and specific factors that are residualized by gASC in the NMS_3sf model; thus, factors from both models are not directly comparable*.* M1: NMS_3sf = Nested Marsh/Shavelson-model of ASC with math ASC, writing ASC, and reading ASC as correlated specific factors; M3: FOCF_4f = First-order correlated factor model with general ASC, math ASC, writing ASC, and reading ASC as factors. | | | | | | | | |
