# Supplementary material for: Profile formation of academic self-concept in elementary school students in grades 1 to 4
Source: PLoS One. 2017 May 18;12(5):e0177854. doi: 10.1371/journal.pone.0177854 (PMC5436832; doi:10.1371/journal.pone.0177854)
Supplement: S1 Appendix — (DOCX) [file pone.0177854.s002.docx]

| **Table B1. Latent Means in Grades 1 to 4 of the Factors Derived from the NMS Model (M1: NMS_3sf) and the First-Order Correlated Factor Model (M3: FOCF_4f), both with Math ASC, Writing ASC, and Reading ASC.** | | | | | |
| --- | --- | --- | --- | --- | --- |
| Grade |  | 1 | 2 | 3 | 4 |
| Model | Factors |  |  |  |  |
|  |  | Reference Group: Grade 1 |  |  |  |
| M1 | gASC | 0 | -.43† | -1.09*** | -1.29*** |
| M3 | gASC | 0 | -.36* | -.73*** | -.77*** |
| M3 | mASC | 0 | -.65† | -1.72*** | -1.74*** |
| M3 | rASC | 0 | -.39 | -1.00*** | -1.20*** |
| M3 | wASC | 0 | -.49* | -.98*** | -1.24*** |
|  |  | Reference Group: Grade 2 |  |  |  |
| M1 | gASC |  | 0 | -.59*** | -.74*** |
| M3 | gASC |  | 0 | -.37*** | -.41*** |
| M3 | mASC |  | 0 | -1.03*** | -1.04*** |
| M3 | rASC |  | 0 | -.58* | -.78*** |
| M3 | wASC |  | 0 | -.50** | -.76*** |
|  |  | Reference Group: Grade 3 |  |  |  |
| M1 | gASC |  |  | 0 | -.08 |
| M3 | gASC |  |  | 0 | -.06 |
| M3 | mASC |  |  | 0 | -.01 |
| M3 | rASC |  |  | 0 | -.20 |
| M3 | wASC |  |  | 0 | -.28 |
| Despite equal nomenclature in M1 and M3, mASC = math ASC, wASC = writing ASC, and rASC = reading ASC are first-order correlated factors in the FOCF_4f model and specific factors that are residualized by gASC in the NMS_3sf model; thus, factors from both models are not directly comparable. M1: NMS_3sf = Nested Marsh/Shavelson model of ASC with math, writing, and reading as correlated specific factors; M3: FOCF_4f = First-order correlated factor model with general ASC, math ASC, writing ASC, and reading ASC as factors. †*p* < .10 **p* < .05 ***p* < .01 ****p* < .001. | | | | | |

Latent mean level differences were estimated in a multigroup model of the scalar invariant model by setting the latent mean in the reference group (i.e., grade level) to zero and estimating the mean in the other group. The estimated latent mean corresponds directly to the mean level difference between grade levels; the significance level indicates whether there is a significant mean difference. Results are presented in Table B1. Table B1 shows that there were significant negative latent mean differences in general ASC between grades 1 and 2 in the FOCF_4f model (*M* = -.36, *p* = .015), and between grades 2 and 3 in both models (NMS_3sf model: *M* = -.59, *p* < .001; FOCF_4f model: *M* = -.37, *p* <.001). Latent mean differences for reading ASC, writing ASC, and math ASC within the FOCF_4f model revealed that writing ASC declined from grades 1 to 2 [*M* = -.49, *p* < .001], whereas math ASC and reading ASC decreased only from grades 2 to 3 [*M* = -.59, *p* = .019]. No further significant changes were found in math ASC, writing ASC, and reading ASC between grades 3 and 4. The pattern of results indicated a lower general ASC, math ASC, writing ASC, and reading ASC at higher grade levels within the first three years of elementary school.
